# Rehabilitation in semantic dementia: Study of effectiveness of lexical reacquisition in three patients

**DOI:** 10.1590/S1980-57642010DN40400009

**Published:** 2010

**Authors:** Mirna Lie Hosogi Senaha, Sonia Maria Dozzi Brucki, Ricardo Nitrini

**Affiliations:** 1 PhD, Speech pathologist, Member of Behavioral and Cognitive Neurology Unit of Department of Neurology, University of São Paulo School of Medicine, São Paulo, Brazil.; 2 MD, Neurologist, Member of Behavioral and Cognitive Neurology Unit of Department of Neurology, University of São Paulo School of Medicine, São Paulo, Brazil.; 3 MD, PhD, Neurologist, Associate Professor, Department of Neurology, University of São Paulo School of Medicine, São Paulo, Brazil.

**Keywords:** semantic dementia, semantic memory, primary progressive aphasia, rehabilitation, lexical reacquisition

## Abstract

Although language rehabilitation in patients with primary progressive aphasia (PPA) is recommended, rehabilitation studies in this clinical syndrome are scarce. Specifically, in relation to semantic dementia (SD), few studies have shown the possibility of lexical relearning.

**Objective:**

To analyze the effectiveness of rehabilitation for lexical reacquisition in SD.

**Methods:**

Three SD patients were submitted to training for lexical reacquisition based on principles of errorless learning. Comparisons between naming performance of treated items (pre and post-training) and non-treated items of the Boston Naming Test (BNT) were made.

**Results:**

All patients improved their performance in naming treated words after intervention. However, decline in performance in naming of non-treated items was observed. Case 1 named zero items at baseline while her performance post-training was 29.4% correct responses without cueing, and 90.7% correct with and without cueing. Case 2 named 6.9% of items correctly at baseline and his performance in post-training was 52.9% without cueing and 87.3%, with and without cueing. Case 3 named zero items at baseline and his performance in post-training was 100% correct responses without cueing. Considering the performance in naming the non-treated items of the BNT, the percentages of correct responses in the first evaluation and in the re-evaluation, respectively were: 16.7% and 8.3% (case 1; 14 month-interval); 26.7% and 11.6% (case 2; 18 month-interval) and 11.6% and 8.3% (case 3; 6 month-interval).

**Conclusions:**

The reacquisition of lost vocabulary may be possible in SD despite progressive semantic deterioration.

## Abstract (pt)

Apesar de recomendada reabilitação da linguagem na afasia progressiva primária (APP), há poucos estudos sobre reabilitação nesta síndrome. Especificamente, quanto à demência semântica (DS), os poucos estudos têm mostrado possibilidade de reaprendizado lexical.

**Objetivo:**

Analisar a eficácia da reabilitação para reaquisição lexical na DS.

**Métodos:**

Três pacientes com DS foram submetidos à reabilitação para reaquisição lexical baseada nos princípios do aprendizado sem erro. Comparações entre desempenhos na nomeação de itens treinados (pré e pós-tratamento) e de itens não-treinados do Teste de Nomeação de Boston (TNB) foram realizadas.

**Resultados:**

Os pacientes obtiveram melhor desempenho na nomeação das palavras treinadas após intervenção. Por outro lado, houve declínio no desempenho dos itens não-treinados. O caso 1 não nomeou nenhum item na linha de base (pré-tratamento) e seu desempenho após o tratamento foi de 29,4% de acertos sem pistas e 90,7% com e sem pistas. O caso 2 nomeou corretamente 6,9% na linha de base e sua performance, pós-treinamento, foi de 52,9% sem pistas e 87,3%, com e sem pistas. O caso 3 não nomeou nenhum item na linha de base e após o treinamento nomeou 100% dos itens sem pista. Considerando a nomeação dos itens lexicais não-treinados do TNB, as porcentagens de acertos na primeira e segunda avaliações foram respectivamente: 16,7% e 8,3% (caso 1; 14 meses de intervalo); 26,7% e 11,6% (caso 2; 18 meses de intervalo) e 11,6% e 8,3% (caso 3; 6 meses de intervalo).

**Conclusões:**

A reaquisição do vocabulário perdido pode ser possível na DS apesar da progressiva deterioração semântica.

Primary Progressive Aphasia (PPA) is a neurodegenerative clinical syndrome in which progressive language impairment occurs in parallel with relative preservation of other cognitive abilities. PPA was firstly defined by Mesulam in 1982 by means of his description of patients who presented gradual deterioration of language in the absence of generalized dementia, and associated to enlargement of the sylvian fissure in the left cerebral hemisphere.^1^

The label “semantic dementia” (SD) was given by Neary et al. (1989) following the reporting of three patients who presented progressive semantic disturbance, evidenced by difficulty in naming and understanding the meanings of words and objects.^2^ The patients presented fluent verbal production, but anomic and difficulties in semantic comprehension, despite the preservation of syntactic comprehension. Later in 1992, Hodges and colleagues characterized the cognitive and linguistic profile for SD in detail, and associated this clinical syndrome to temporal lobe atrophy.^3^ In 1998, a consensus on clinical diagnostic criteria for frontotemporal lobar degeneration (FTLD) was published. In this consensus, Neary et al.^4^ proposed that SD, as one of the three most frequent clinical syndromes in FTLD, be defined as “semantic aphasia associated to the associative agnosia”. Adlam et al. (2006)^5^ subsequently suggested the redefinition of associative agnosia issues in SD, pointing out that the non-verbal semantic impairment tends to be much less prominent than the verbal semantic deficit. The researchers proposed the substitution of the use of term “associative agnosia” to characterize SD, by “impairment on tests of non-verbal associative knowledge”.

Recently, Rogalski & Mesulam and Mesulam & Weintraub published a new revision of the diagnostic criteria for PPA, outlining the core, ancillary and exclusionary criteria for PPA.^6,7^ According to the authors, the clinical profile of PPA can be defined as “an aphasic dementia where the language impairment emerges in relative isolation and is the major determinant in the limitation of daily living activities. Perception, memory, personality are relatively preserved during the initial 1-2 years”. Specifically, in relation to the different subtypes of PPA, the authors suggested classification into three main clinical subtypes as proposed by Gorno-Tempini et al.:^8^ agrammatic subtype, logopenic progressive aphasia, and semantic subtype. These variants are the main subtypes, but other clinical manifestations of PPA are described in the literature. According to Mesulam and Weintraub,^7^ the semantic subtype fits the criteria for semantic dementia redefined by Adlam et al.^5^.

Considering that PPA patients present selective impairment and are independent regarding daily living activities, except in situations which rely heavily on linguistic abilities, cognitive-linguistic rehabilitation has been recommended.^7,9-14^ Rehabilitation plays a very important role in minimizing the impact of the linguistic disturbance in daily life. Although rehabilitation is recommended, published studies addressing rehabilitation in this clinical syndrome are scarce.

More specifically, in relation to the patients with SD, the few intervention studies available have shown the possibility of lexical relearning despite progressive semantic deterioration. To date, only 12 SD patients submitted to lexical reacquisition rehabilitation have been reported.^15-24^ Snowden & Neary (2002) studied two cases with SD, each submitted to different experiments, and showed the possibility of relearning of lost words.^15^ According to the authors, the study showed the importance of medial temporal lobe structures for the acquisition of semantic facts. Graham et al. discussed the reacquisition of vocabulary from self-training carried out by patient D.M. with SD.^16-17^ These authors compared this case with another (A.M.) who did not enjoy the same success in lexical reacquisition. Frattali (2004) described a case in which the process of lexical relearning of a list of nouns and verbs was carried out in conversational situations.^19^ The patient presented improvement after the period of therapy, but had not retained the gains at the next follow-up (three months). Jokel et al. (2006) studied a treatment program (home practice) in only one case with SD and evidenced the benefit of the program for trained lexical items.^18^ In 2009, Heredia et al. published the case study of patient C.U.B., and found, in contrast to the other cases, a possibility of generalization of lexical relearning.^22^ In addition, the authors had also observed the maintenance of relearning despite intense deterioration of semantic memory. Jokel et al. (2010) published another SD case which was successfully submitted to treatment for anomia based on principles of the errorless learning approach using a computer program.^20^ Newhart et al (2009) compared the benefits of rehabilitation in two PPA cases, one with logopenic progressive aphasia, and the another with SD. Both presented improvement in naming, but the generalization was evidenced only in the patient with logopenic progressive aphasia.^21^ Robinson et al. (2009) verified the effectiveness of treatment for object naming, definition and object use in two SD cases. Both patients showed improvement, but only one had retained the improvement at the follow up.^23^ Bier et al. (2009) studied the method of formal-semantic therapy (which focuses on restoring lost concepts and discriminating between these concepts) used in non-degenerative fluent aphasics combined with the spaced retrieval method in an SD case. The authors observed the possibility of improvement in naming performance, but with limitations.^24^

Considering the few studies on intervention in SD and the fact that most of these display some benefits for the patients, we can conclude that rehabilitation contributes toward minimizing linguistic difficulties, and may consequently postulate that rehabilitation can also contribute to improving the life quality of patients and their relatives.

Given the importance of rehabilitation in SD and the lack of studies in the literature, the present study sought to report the lexical relearning training of three SD cases submitted to language rehabilitation. The therapeutic approach was based on previous studies that showed the possibility of lexical reacquisition in SD patients. In other words, the purpose of this study was to analyze the effectiveness of rehabilitation for lexical reacquisition in three patients with SD.

We raised two hypotheses, the first based on previous studies that patients with SD have the capacity to reacquire lost lexical items. The second hypothesis was that patients will present improvement only on the reacquisition of trained lexical items, and this would not be generalized for non-treated lexical items. This notion was based on the characteristics of the semantic disturbance of SD: the anomia in SD occurs mainly due to semantic degradation and this leads to systematic semantic errors.

## Methods

### Subjects

**Figure 1:**
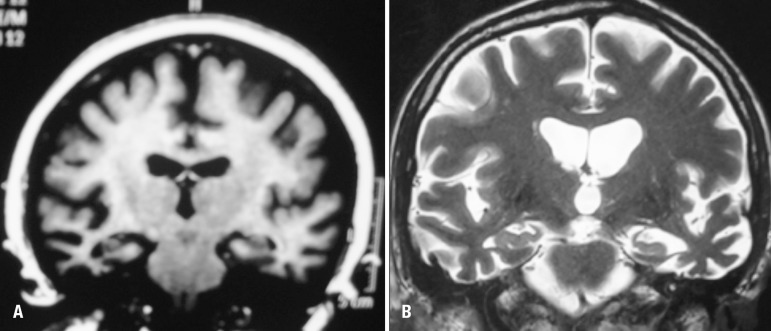
Examples of structural imaging (MRI) of cases 1 and 3. [A] Case 1. Anterior temporal lobe atrophy, more prominent on right side; [B] Case 3. Anterior temporal lobe atrophy, more prominent on left side.

Three SD patients were submitted to cognitive-linguistic rehabilitation. The demographic data of these cases are presented in Table 1. The group comprised two men and one woman aged between 55 and 77, with an educational level greater than 11 years. Table 2 shows the performances of the three patients on language tests and semantic memory tasks. Some of these tasks had been described in a previous study.^25,26^ As expected for the profile of SD clinical syndrome, all the cases presented low performance in visual confrontation naming (Boston Naming Test - BNT), difficulties on the word definition task and oral word comprehension (word-picture matching task), preserved syntactic comprehension, surface dysgraphia and impairment on tests of non-verbal associative knowledge. The diagnosis of SD was confirmed by experienced cognitive neurologists and was based on clinical history, neurological examination, neuropsychological and language evaluation, and neuroimaging data. Results on neuroimaging examination (MRI) were consistent with diagnoses of SD. All the cases presented temporal lobe atrophy. Case 1 presented anterior temporal lobe atrophy, which was more prominent on the right side whereas cases 2 and 3 presented temporal lobe atrophy predominantly in the left hemisphere (Figure 1)

**Table 1:** Demographic data of semantic dementia patients.

	Case 1	Case 2	Case 3
Age	55	77	56
Age of onset	53	76	54
Gender	F	M	M
Educational level	11	16	16

**Table 2:** Performance on language and semantic memory tasks.

	Case 1	Case 2	Case 3
Syntactic comprehension - Simple and complex sentences*	37/38	38/38	38/38
Semantic comprehension - Words^+^	56/90	58/90	68/90
Repetition - Words and non-words* - Sentences (high frequency)^§^ - Sentences (low frequency)^§^	25/25 7/8 3/8	25/25 8/8 7/8	25/25 7/8 4/8
Reading aloud - Words and non-words^||^	86/110surface dyslexia	109/110semantic dyslexia	96/110surface dyslexia
Writing to dictation - Words and non-words^||^	28/46surface dysgraphia	70/110surface dysgraphia	62/110surface dysgraphia
Visual confrontation naming - Pictures^¶^	10/60	16/60	7/60
Verbal fluency - Animals - FAS (total)	520	613	617
Word definition^+^	2/13	4/13	3/13
Visual sorting^+^	137/168	150/168	155/168

* Beta MT-8630;

+ Semantic memory battery;

§ Boston Diagnostic Aphasia Examination^31^;

|| HFSP protocol^32^;

¶ Boston Naming Test^33^.

### Stimulation for lexical reacquisition

The patients were submitted to language rehabilitation individually. One of the principle focuses was the stimulation for lexical reacquisition since the main complaint of the patients and their relatives was related to verbal semantic impairment (anomia and word comprehension impairment), the most evident characteristics in SD.

The stimulation for the lexical reacquisition carried out was based on principles of the errorless learning approach.^27-29^ For the training of lexical reacquisition, it was necessary to carry out a selection of words for stimulation. The selection of words to be trained was personalized, taking into account the difficulties and particular needs of each patient and their relevance to daily life.

The selection of the words occurred in a dynamic way and in several different manners:

[1] from the observation of the patients’ lexical problems in situations involving spontaneous speech;
[2] through information and complaints provided by the patients who wrote down the lexical difficulties faced in their daily lives;
[3] through information supplied by the relatives.

**Figure 2:**
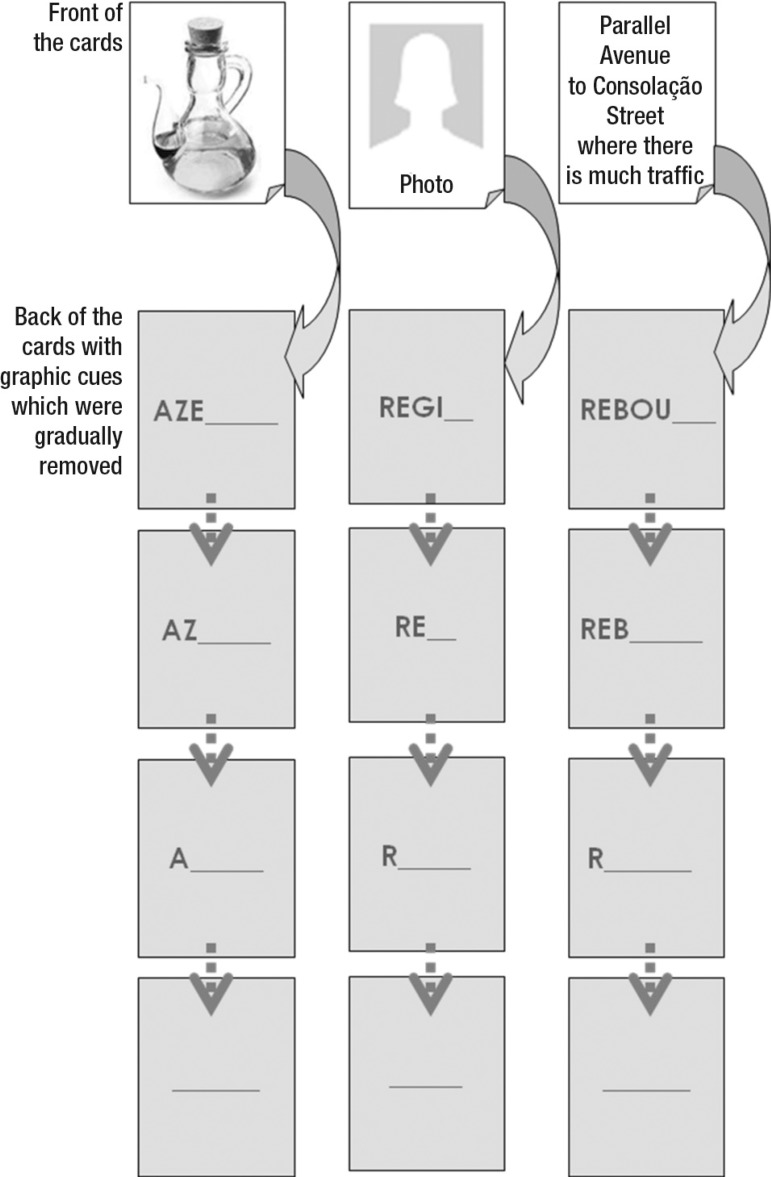
Cards samples for training of lexical reacquisition.

Using the selection of the words to be trained, cards were assembled containing figures, photos or written descriptions on one side of the cards while the back of each card contained a written graphic cue that induced the correct naming of each stimulus. With the training and progression in correct answers in the naming with the aid of the cues, the letters of the graphic cues were gradually removed as shown in Figure 2 (vanishing cues).

Figure 2. Cards samples for training of lexical reacquisition.

The sessions of this lexical reacquisition intervention were carried out once or twice a week. All the patients were instructed to carry out daily home self-training. Despite the recommendation of daily training, case 2 carried out the training only once or twice a week.

For the analysis of the effectiveness of the training of lexical reacquisition, comparisons between naming of the treated items (pre and post-training) and of non-treated items (BNT) were carried out for each case. The length of the training and the number of treated lexical items varied for each patient (case 1: 119 treated items, 14 months of intervention; case 2: 87 treated items, 18 months of intervention; case 3: 65 treated items, 6 months of intervention). The assessment of non-treated items of BNT - first evaluation and re-evaluation - occurred in parallel at the beginning and end of the lexical stimulation in each case.

## Results

**Figure 3:**
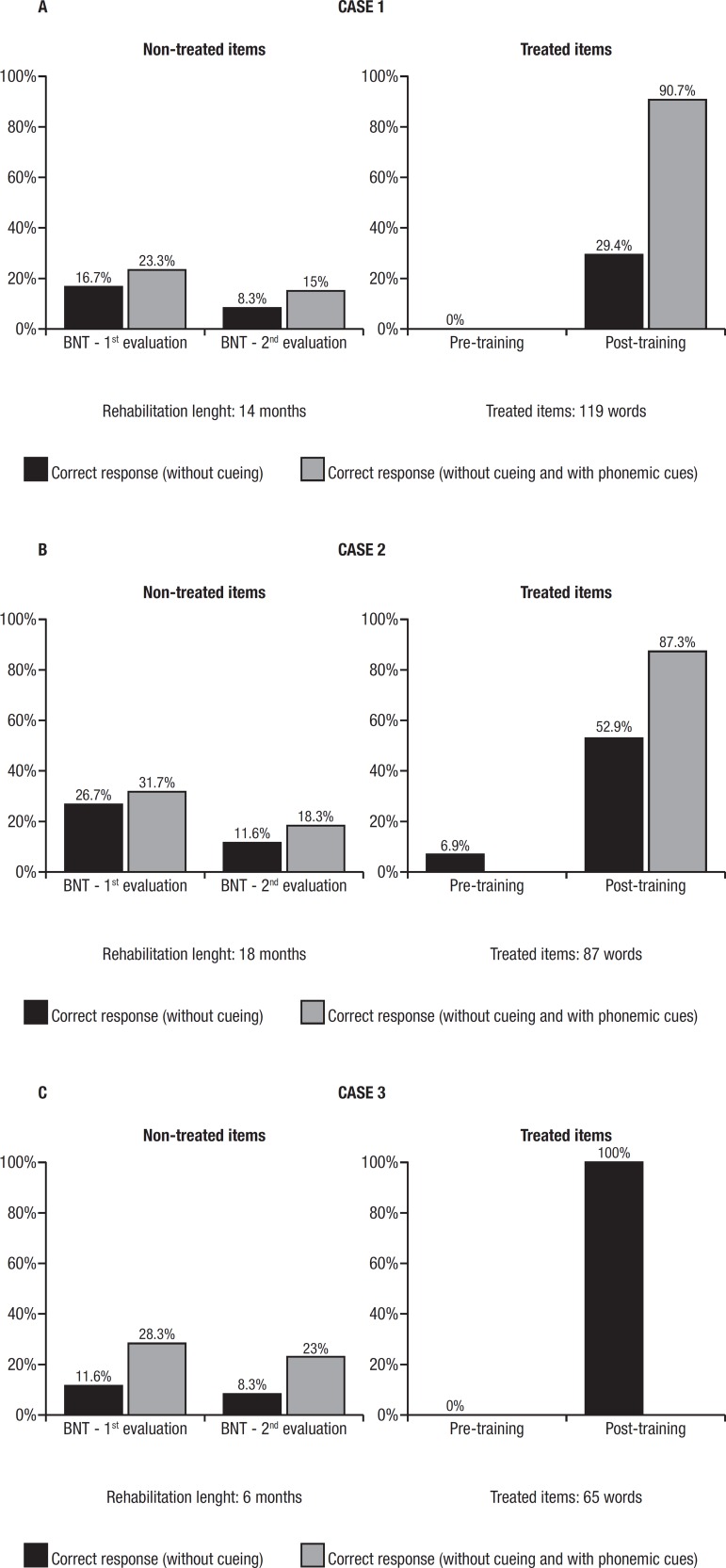
Comparison of patient’s performance in the naming of non-treated and treated lexical items.

The results of the patients’ performances on the naming of non-treated lexical items and treated items are depicted in Figure 3. Each sub item of Figure 3 shows the results for each patient displayed on two graphs: the first graph shows the comparison of the performances on the naming of non-treated items (Boston Naming Test) for the first and the second evaluations that occurred at the beginning and end of the stimulation of lexical reacquisition. The second graph shows the comparison of the performances on the naming of the treated items (pre and post-training).

All three patients presented a decline in performances in naming non-treated items between the first evaluation and the re-evaluation yet presented improvement in the performances in naming of treated items (pre and post-training).

## Discussion

All the patients showed improved performance in naming treated items after rehabilitation, in spite of decline in performance on non-treated items. This data indicates the effectiveness of the lexical stimulation without generalization. Despite the absence of generalization, the possibility of patients using the reacquired lexical items functionally, i.e. in “extra-training” situations, was demonstrated in spontaneous speech situations.

These results confirm the two hypotheses put forward initially: firstly, that SD patients have the capacity to relearn lost lexical items, and secondly that the reacquisition only occurs for treated lexical items, without generalization for non-treated lexical items.

In SD, anomia occurs principally due to semantic deterioration rather than lexical access impairment. For this reason and because of the type of therapeutic intervention used for lexical reacquisition, generalization of the lexical relearning of non-treated items did not occur. This non-generalization for non-treated lexical elements was also evidenced in other studies.^16-18,21^

The benefits of the rehabilitation process involving the lexical reacquisition of our three patients varied from case to case. Case 3 was the patient who presented the greatest gain as he was able to name all treated items without cues after the intervention. In this study, it was not possible to associate the intensity of the therapeutic benefit to factors such as age, gender, rehabilitation time and severity of the disturbance. Case 3 had the greatest gain but presented the lowest performance on the Boston Naming Test in the first evaluation and underwent less rehabilitation in terms of time.

The success of the therapeutic intervention, even with limitations in neurodegenerative disease such as SD, is relevant to patients and their relatives when minimizing linguistic difficulties. Moreover, it serves as a motivational stimulus, as patients and relatives have the opportunity to experience the possibility of relearning, reinforcing one of the characteristics of PPA - the presence of a selective disturbance along with relative preservation of other cognitive abilities.

The personalized selection of words to be trained was important, by considering the difficulties and particular needs of each patient and relevance in their daily lives.

Akin to previous studies on non-pharmacological intervention in SD, the present study has the limitation of a small number of cases in the sample. Another important issue is, although the training for the three patients had the same theoretical principles of errorless learning, there was no strict use of the same lexical items or number of sessions for the three cases. In contrast to other studies, we chose to personalize the selection of words to be trained according to the particular needs of each patient. This option can be viewed as a disadvantage for the comparison of variables and those involved in the effectiveness of the treatment study. However, we believe this to be important for reaching the individuals’ needs and for motivational aspects.

In spite of these limitations, the results have proven the effectiveness of the intervention for lexical reacquisition in the three SD cases, despite progressive semantic deterioration, thus demonstrating the ability of lexical relearning in SD patients.
